# Supplementary material for: Content-rich biological network constructed by mining PubMed abstracts
Source: BMC Bioinformatics. 2004 Oct 8;5:147. doi: 10.1186/1471-2105-5-147 (PMC528731; doi:10.1186/1471-2105-5-147)
Supplement: Additional File 5 — The original Chilibot query results of the term "long-term potentiation (LTP)" and 22 other terms, limiting the latest references analyzed to the years 1990, 1995, 2000, and 2004. [file 1471-2105-5-147-S5.bz2 › chilibotAdditionalFile5/ltp2004/html/CAMKII_ACTIN.html]

 


 **CAMKII** and **ACTIN** 
  
Found 22 abstracts in PubMed,  **22 abstracts were retrieved and analyzed**.  


---

 Search Google  |
 PDF files only 
|  EDU domain only 

---

**Interactive relationship** (e.g. stimulation, inhibition, etc)

- KN62 an inhibitor of  **CaMKII**  and CaMKIV attenuated c fos, brain natriuretic peptide, alpha skeletal  **actin** , and atrial natriuretic peptide expression.  Ref: 11073891 Circ Res, 2000
- The results suggest that beta1 integrin is tyrosine phosphorylated and links with  **actin**  via alpha actinin in HBE cells but prevented from linking with  **actin**  in MCF 7 cells by phosphorylation at both tyrosine and serine threonine of beta1 integrin which forms a complex with alpha actinin and  **CaMKII** .  Ref: 11716783 BMC Cell Biol, 2001
- **CaMKII**  was also localized to the subapical cytoplasm and was clearly displaced following  **actin**  filament disruption.  Ref: 12893633 Am J Physiol Gastrointest Liver Physiol, 2003
- We also suggest how recently discovered binding interactions could provide a structural readout mechanism the autophosphorylated state of  **CaMKII**  binds tightly to the NMDAR and forms, through  **CaMKII**  actinin  **actin**  4.1 SAP97 linkages, additional sites for anchoring AMPARs at synapses.  Ref: 11502252 Neuron, 2001
- Although F  **actin**  dissociation into the cytosol required autophosphorylation of or calcium calmodulin binding to beta  **CaMKII** , PSD translocation required binding of calcium calmodulin to either the alpha or beta  **CaMKII**  subunits.  Ref: 10102820 Science, 1999
- In contrast, the synaptic clustering of calcium calmodulin dependent protein kinase II  **CaMKII**  alpha, another core component of the PSD, was completely dependent on an intact  **actin**  cytoskeleton and was partially disrupted by detergent.  Ref: 10844024 J Neurosci, 2000
- PKC induced accumulation of  **CaMKII**  was dependent on filamentous  **actin** .  Ref: 11896155 J Neurosci, 2002

**Parallel relationship** (e.g. studied together, co-existance, homology, etc.)

- Here, we use green fluorescent protein tagged GFP tagged  **CaMKII**  isoforms and show that CaMKIIbeta is bound to F  **actin**  in dendritic spines and cell cortex while CaMKIIalpha is largely a cytosolic enzyme.  Ref: 9768845 Neuron, 1998
- Postsynaptic densities PSD from excitatory synapses have yielded many cytoskeletal proteins including  **actin** , spectrin, tubulin, microtubule associated proteins and calcium calmodulin dependent protein kinase II  [ **CAMKII** ] .  Ref: 9670221 Prog Neurobiol, 1998
- ... dispersal of NMDA receptors occurred by a different mechanism independent of  **actin**  or  **CaMKII** .  Ref: 11896155 J Neurosci, 2002
- It is shown that N methyl D aspartate NMDA receptor stimulation reversibly translocates green fluorescent protein tagged  **CaMKII**  from an F  **actin**  bound to a PSD bound state.  Ref: 10102820 Science, 1999
- We here demonstrate for the first time the participation of CaM,  **CaMKII**  and the  **actin**  cytoskeleton in the regulation of Gly transport in glia.  Ref: 11841571 J Neurochem, 2002
